# It Takes Time: Vigilance and Sustained Attention Assessment in Adults with ADHD

**DOI:** 10.3390/ijerph19095216

**Published:** 2022-04-25

**Authors:** Anselm B. M. Fuermaier, Lara Tucha, Nana Guo, Christian Mette, Bernhard W. Müller, Norbert Scherbaum, Oliver Tucha

**Affiliations:** 1 Department of Clinical and Developmental Neuropsychology, Faculty of Behavioral and Social Sciences, University of Groningen, 9712 TS Groningen, The Netherlands; lara.tucha@med.uni-rostock.de (L.T.); n.guo@rug.nl (N.G.); 2 Department of Psychiatry and Psychotherapy, University Medical Center Rostock, 18147 Rostock, Germany; oliver.tucha@med.uni-rostock.de; 3 Department of Psychology, Protestant University of Applied Sciences Bochum, 44809 Bochum, Germany; mette@evh-bochum.de; 4 Department of Psychiatry and Psychotherapy, Faculty of Medicine, University of Duisburg-Essen, 45147 Essen, Germany; bernhard.mueller@uni-due.de (B.W.M.); norbert.scherbaum@uni-due.de (N.S.); 5 Department of Psychology, University of Wuppertal, 42119 Wuppertal, Germany; 6 Department of Psychology, National University of Ireland, W23 F2H6 Maynooth, County Kildare, Ireland

**Keywords:** adult ADHD, neuropsychology, vigilance, sustained attention, impairment

## Abstract

**Objectives**: The present study compares the utility of eight different tests of vigilance and sustained attention in the neuropsychological examination of adults with Attention-deficit/hyperactivity disorder (ADHD). **Methods**: Thirty-one adults diagnosed with ADHD performed eight tests for vigilance and sustained attention, spread over three assessment days. **Results**: Adults with ADHD showed cognitive impairments in most tests and test variables, even though their sensitivity differed greatly. No specific type of test variable stands out to be the most sensitive, and no evidence for a differential deterioration of performance over time was observed. **Conclusion**: This study underscores the role of vigilance and sustained attention tests in the assessment of adult ADHD. It is further concluded that summary scores over the entire test duration are sufficient, but that all variables of a test should be considered. Finally, we hypothesize that reassessment on a different day may benefit a more accurate clinical assessment of adults with ADHD, in order to adequately take intraindividual fluctuations and limitations regarding test reliability into account.

## 1. Introduction

Attention-deficit/hyperactivity disorder (ADHD) [1] in adulthood is associated with cognitive impairment in various aspects of attention and executive control [2], which adversely affect individuals’ daily life functioning and requires thorough clinical assessment and treatment. Despite neuropsychological assessment not being essential for the diagnosis of ADHD, it is often indicated in order to objectify and characterize individual cognitive strengths and weaknesses, guide treatment planning, support treatment evaluation, and increase individual compliance and adherence to treatment [3,4].

Vigilance and sustained attention tests are among the most sensitive measures to reveal cognitive impairments in adults with ADHD, as demonstrated by a large body of empirical research on ADHD in children [5,6], adolescents and adults [7,8,9,10], as well as older adults [11]. In addition, the prominent role of vigilance and sustained attention assessment in the clinical evaluation of adult ADHD was also stressed in a consensus report by an international expert panel in a recent Delphi study [12]. Sustained attention refers to the maintenance of attention and concentration over a long and unbroken period of time [13]. The assessment of sustained attention plays an important role in a neuropsychological evaluation, as intact sustained attention is crucial for successful daily functioning, such as driving a vehicle, following and contributing to conversations, or maintaining adequate performance over a workday. Although often used interchangeably, vigilance differs from sustained attention as vigilance is characterized by sustained attention to monotonous stimulus configurations and infrequent response demanding cues. Tests for sustained attention and vigilance are popular instruments in research and clinical practice, as they were shown to unfold symptoms of distractibility, mind wandering, and deficits in inhibitory control (impulsivity), reflected in test variables, indicating slow responses, a larger variability in reaction times, as well as errors of omission and commission. It has been argued that disturbances in sustained attention and vigilance are specifically indicated by a greater deterioration in performance over time (time-on-task effects; TOT; [14]). Thus, a reasonably long period of testing time may be necessary before a significant decrement in performance becomes evident [15]. This notion is supported by findings showing executive dysfunctions but intact attention performance in young adults with ADHD, when short-term attention tasks are administered that take only a few minutes to perform (e.g., the d2-test for attention as presented by Fabio and Capri [16,17]). It therefore remains unknown whether it is the features of sustained attention and vigilance tasks that are responsible for their clinical value, or whether it is the task duration. Further, interactions with disease characteristics (e.g., symptom presentations) may explain the mechanisms behind how neuropsychological tasks unfold their clinical value [16].

The neuropsychological evaluation of sustained attention and vigilance is commonly accomplished by variants of the continuous performance test (CPT; [18]). With the support of test publishers and clinical and scientific partners, a broad variety of CPTs have become available on the market that are implemented worldwide in routine clinical practice and research for the assessment of vigilance and sustained attention. Examples of the most commonly used tests are the Cambridge Neuropsychological Test Automated Battery (CANTAB) [19], the Conners Continuous Performance Test (Conners’ CPT) [20], the Integrated Visual and Auditory Continuous Performance Test (IVA-2) [21], vigilance tests of the Vienna Test System (VTS, i.e., VIGILANCE (VIGIL) and Perception and Attention Functions - Vigilance (WAFV)) [22], the Test Battery of Attention Performance (TAP) [23], and the Test of Everyday Attention (TEA) [24], or the Test of Variables of Attention (TOVA) [25]. Although all tests share core features of vigilance and sustained attention, there are pronounced differences between these tests, e.g., in test design (e.g., type, number, and arrangement of stimuli), test duration (7–33 min), stimulus mode (e.g., auditory or visual stimulus channel), and outcome variables (e.g., response time and response time variability, omission and commission errors, index scores).

While the availability of a variety of tests is welcome, it is currently difficult to select a specific test for the assessment of an adult with ADHD, as these tests and test performances of adults with ADHD have not been compared yet. The nature of relatively long test durations complicates studies on the comparison of the utility of the various vigilance and sustained attention tests on the same sample of participants, as only a limited number of tests can be performed in one assessment session without fatigue effects interfering with test performance. A comparison of the sensitivity of the various tests would benefit clinical practice and research, in order to gain a more thorough understanding of the nature of cognitive impairment in adult ADHD, facilitate test selection in clinical assessment and research, and examine the robustness of vigilance and sustained attention deficits in adults with ADHD.

For the present study, we designed a test battery that is composed of eight vigilance and sustained attention tests, that are all internationally renowned and accepted and routinely applied in clinical practice and research. This test battery was administered to a sample of 31 adults who underwent a thorough clinical assessment and were all diagnosed with ADHD. The assessment with vigilance and sustained attention tests was spread over three assessment sessions on different assessment days. This study aims to (1) determine the most sensitive vigilance and sustained attention tests and test variables for the detection of cognitive impairment in adult ADHD and (2) explore the robustness of vigilance and sustained attention impairments by examining the congruence between the test and test variables. This study further aims to (3) explore the time-on-task effects of adults with ADHD and learn about potential sustained attention deficits in this population.

## 2. Materials and Methods

### 2.1. Participants

Thirty-one adult patients diagnosed with ADHD took part in this study. All patients were referred to the ADHD outpatient clinic of the Department of Psychiatry and Psychotherapy, University of Duisburg-Essen, Germany, for a diagnostic assessment. The individuals underwent a comprehensive diagnostic assessment by trained psychologists or psychiatrists. The diagnosis of ADHD was established based on the criteria as outlined in the Diagnostic and Statistical Manual of Mental Disorders, 5th Edition (DSM–5) [1]. The assessment procedure included semi-structured interviews to evaluate ADHD psychopathology (i.e., the Wender–Reimherr Interview [26] and the Essen-Interview-for-schooldays-related-biography [27]). Furthermore, the diagnostic assessment included symptom self-reporting scales to quantify the retrospective and current ADHD symptom severity [28]. The German version of the Wender Utah Rating Scale (WURS-K) was used to evaluate retrospective ADHD symptoms in childhood [29,30], while the Conners’ Adult ADHD Rating Scale (CAARS) was administered to assess current ADHD symptoms [31]. The Beck Depression Inventory (BDI–2) [32] was used to identify and quantify depressive symptoms, as depression and depressive symptoms occur commonly in patients with ADHD. Self-reported impairments in various domains of daily living were assessed with the Weiss Functional Impairment Rating Scale (WFIRS, Canadian ADHD Resource Alliance [33]). The diagnostic evaluation also included objective measures such as evidence derived from school reports and reports of failure in academic and/or occupational achievement, and comprised multiple informants for all individuals (e.g., employer evaluation, partner or parent-reports). Furthermore, 12 of the 31 patients with ADHD showed evidence for one or more psychiatric disorders other than ADHD (comorbidities), including mood disorders (*n* = 11) and anxiety disorders (*n* = 1). Twenty-nine of the 31 patients with ADHD were prescribed stimulant medication at the time of the assessment, but withdrew from medication intake at least 48 h prior to each of the three neuropsychological assessments. Table 1 presents patients’ characteristics and symptom scores.

### 2.2. Materials

#### 2.2.1. Questionnaires and Rating Scales for Symptoms and Impairments

The German short version of the Wender Utah Rating Scale (WURS-K [29]) was administered to all participants to quantify self-reported retrospective ADHD symptoms. The WURS-K consists of 25 items using a 5-point Likert scale ranging from 0 (*not at all or very slightly*) to 4 (*very much*). The participants were instructed to rate each item based on their recall of childhood experiences. A sum score was calculated, which indicates the severity of retrospective symptoms of ADHD.

The self-reported, long version, of the Conners’ Adult ADHD Rating Scales (CAARS [31]) was used to measure the presence and the severity of current ADHD symptoms. This scale includes 66 items rated on a 4-point Likert scale ranging from 0 (*not at all/never*) to 3 (*very much/very frequently*). Sum scores can be calculated for eight subscales, however, for the present study only the DSM subscales for inattention, hyperactivity/impulsivity, and the total ADHD symptoms were used.

The Weiss Functional Impairment Rating Scale—Self-Report (WFIRS—S [33]) is a measure specifically developed to assess impairments commonly occurring in patients with ADHD. The WFIRS—S comprises 70 items on a 4-point Likert scale ranging from 0 (*never or not at all*) to 3 (*very often or very much*). In addition, Not Applicable (*N/A*) is an additional option to answer items that do not apply to participants. Participants were asked to rate each item based on their experiences in the last month. Items are divided into seven specific domains, including Family (8 items), Work (11 items), School (11 items), Life skills (12 items), Self-concept (5 items), Social (9 items), and Risk (14 items). A mean score was calculated for each domain by dividing the sum score by the number of items, excluding the items rated Not Applicable (*N/A*) from the total number.

The German version of the Beck Depression Inventory-II (BDI-II [32]) was used to assess the existence and severity of depression symptoms. The BDI-II is a self-report inventory, including 21 items that are rated on a 4-point Likert scale based on their experiences in the past two weeks. A sum score indicating the severity of depression symptoms was calculated for the scale.

#### 2.2.2. Neuropsychological Tests of Vigilance and Sustained Attention

##### Test of Everyday Attention (TEA)

The TEA is a non-computerized test that was designed to comprehensively assess different aspects of attention [24]. One of the subtests of the TEA, i.e., elevator counting subtest, was used in the present study to assess vigilance performance. In this subtest, participants were required to imagine they were in an elevator and the visual floor-indicator light was broken. During the test, a series of tones that resembled the tone of an elevator passing each floor were presented, which can help participants to indicate which floor they were on. Participants were asked to tell which floor they were on when the voice “how many” appeared. The number of correct responses was recorded. The maximum score is 7. The test manual reports that none of the normative sample made more than one error out of seven on this subtest and indicated that any score equal to or less than 5 indicates impaired performance.

##### VIGIL

The VIGIL is a computerized test of the Vienna Test System (VTS [22]) for the assessment of vigilance. During this test, an illuminated dot moves along a circular path in small jumps. In most cases, the dot jumps to the next circle in a step-wise progression. Occasionally, the dots make a double jump (targets). When double jumps occur, the participants were requested to press the response button as quickly as possible. This test includes 1000 steps in total, 32 of which are double jump steps (target rate 3.2%). The test duration is about 33 min. The outcome measures include the number of correct responses, the number of commission errors, and the mean reaction time (RT) of correct responses.

##### Perception and Attention Functions—Vigilance (WAFV)

The WAFV is a vigilance test of the VTS [22]. The visual, long-form of the WAFV consists of 900 squares presented consecutively on a computer screen. Participants were required to press the response button as quickly as possible when a square became darker (target). The frequency of targets is 5% of the total number of stimuli. The administration time of this test is 30 min. The outcome measures include the number of omission and commission errors, the mean RT of correct responses in milliseconds, and the logarithmic standard deviation of the reaction time (SDRT). Additionally, these outcome measures were compared between the first- and second-half of the test to indicate the time-on-task effects, by calculating the ratio of the performance of the second-half by the first-half.

##### Test Battery for Attention Performance—Vigilance (TAP)

The TAP is a computer-based assessment of various aspects of attention [23]. This study made use of the visual test condition of a “moving bar” in the vigilance test of the TAP. In this subtest, a light-colored bar moving up and down was presented on the screen. The deflection of the vertical movements from the center varies in its extent. On rare occasions, the upward deflection is much larger (targets). Participants’ task was to press the response button as quickly as possible when the target appeared (i.e., when the bar made a significantly larger upward deflection). A total of 2800 stimuli (with 36 targets) were presented. This test takes about 30 min to complete. The outcome measures include the number of omission and commission errors, the median RT of correct responses in milliseconds, and the SDRT. Additionally, these outcome measures were compared between the first- and second-half of the test to indicate the time-on-task effects, by calculating the ratio of the performance of the second-half by the first-half.

##### Test of Variables of Attention (T.O.V.A.)

The T.O.V.A. is a computerized CPT that was developed to assess the key components of attention [25]. In this test, two kinds of squares flashing on the screen were presented to participants one by one. The only difference between the two different kinds of squares was the location of a small hole in the square; one kind of square had a small hole near the top and another square had a small hole near the bottom. Squares with a hole near the top were defined as targets and participants were asked to press the response button as fast and accurately as possible when targets appeared. The first-half of the test is defined as the target infrequent condition and includes 72 targets and 252 non-targets (22% target rate). The second-half of the test is defined as the target frequent condition and includes 252 targets and 72 non-targets (88% target rate). The test takes about 22 min to complete. The outcome measures include the number of omission and commission errors, the mean RT of correct responses in milliseconds, and the SDRT. Additionally, these outcome measures were compared between the first- and second-half of the test to indicate the time-on-task effects, by calculating the ratio of the performance of the second-half by the first-half.

##### Conners Continuous Performance Test—3rd Edition (Conners CPT 3)

The Conners CPT 3 is a task-oriented computerized visual assessment of attention-related problems [20]. During the test, participants were presented with a series of letters appearing one by one on the monitor, and were asked to respond to any letter by pressing the spacebar, excepting the letter “X”. A total of 360 stimuli (80% targets) were presented, with inter-stimulus intervals (ISI) of one, two, or four seconds. Each stimulus was presented for 250 milliseconds. The Conners CPT 3 is divided into six blocks and the ISIs are counterbalanced between blocks. The administration time of this test is 14 min. The outcome measures include the number of omission and commissions errors, the mean RT of correct responses in milliseconds, and the SDRT. Time-on-task effects are shown by the block change of the mean RT, which is the slope of change in the mean RT across blocks, indicating the change in the mean response speed across blocks.

##### Cambridge Neuropsychological Test Automated Battery—Rapid Visual Processing (CANTAB—RVP)

The CANTAB—RVP is a computer-based visual test of vigilance [19,34]. In this test, participants were presented with numbers (from 2 to 9) appearing one at a time in the center of the computer screen and were asked to press the response button whenever they spotted a target sequence (i.e., 2-4-6, 3-5-7, or 4-6-8). For example, a target sequence “2-4-6” is shown by a ‘2’, immediately followed by a ‘4’, and immediately followed by a ‘6’. Participants were asked to press the response button only when they saw the last number of a target sequence (e.g., ‘6’ of the “2-4-6” sequence). A total of 300 stimuli were presented, including 27 targets (9%). The test duration is around 6.5 min. Outcome measures include the probability of hit (p(hit)), the number of commission errors, and the mean RT of correct responses in milliseconds. The p(hit) refers to the probability of correct response and is calculated by dividing the number of correct responses by the sum of the number of correct responses and missing responses (hits/(hits + misses)).

##### Integrated Visual and Auditory Continuous Performance Test (IVA-2)

The IVA-2 is a computerized task of attention [35]. In this test, a series of “1”s and “2”s were presented to participants in a pseudo-random combination of visual and auditory stimuli. Participants were required to respond as quickly as possible whenever they saw or heard a “1” (target) and not to respond whenever they saw or heard a “2” (foil). The IVA-2 consists of five sets of two blocks each, and each block includes 50 trials. The number of visual and auditory stimuli is equal in each block. The target stimulus rate (“1”) is 84% for the first and 16% for the second block of each set. The administration time of this test is about 15 min. The outcome measures include the number of omissions, number of commissions, the mean RT of correct responses in milliseconds, the SDRT, and the stamina. Stamina indicates the time-on-task effects and is calculated by the mean RT of the first two sets divided by the mean RT of the last two sets.

#### 2.2.3. Procedure

After completion of the diagnostic assessment, all individuals with ADHD were invited to take part in the research study on a voluntary basis. Participation in this study included three neuropsychological assessments, each taking about 60–90 min, on three different days, and each being administered in the rooms at the Department of Psychiatry and Psychotherapy of the LVR Hospital Essen, Germany. The three assessments per participant were scheduled in three different weeks, each time on the same day of the week and time point (i.e., morning, noon, afternoon). Participants were requested to perform eight vigilance tests, but no more than two to three tests per assessment, and with sufficient breaks in between each test. The test order was randomized across participants to avoid confounding influence of practice effects or fatigue. As the neuropsychological assessment with vigilance tests was not part of the routine clinical examination, patients with ADHD were compensated with a financial reward (60 Euros) upon completion of all three assessments. Further, participants agreed to their routine clinical information (e.g., diagnostic status, medication intake, etc.) being used for scientific purposes. It was stressed to all patients that taking part in the research project was independent of their clinical trajectory and did not affect their clinical assessment and treatment. However, patients with ADHD who were prescribed stimulant drug treatment agreed to withdraw from intake of stimulant medication at least 48 prior to each neuropsychological assessment. The study was conducted under the guidelines of the latest version of the Declaration of Helsinki and was approved by the local ethical review board of the medical faculty of the University of Duisburg-Essen, Germany.

#### 2.2.4. Statistical Analysis

Vigilance and sustained attention performance scores are presented in descriptive statistics for all individuals. Further, test performance scores are indicated by *T*-scores based on normative data, as presented in the respective test manuals. *T*-scores are also inspected to indicate the number of individuals with ADHD showing impairment in the respective aspects of functioning, i.e., defined as impairment if an individual score is equal to or lower than one standard deviation below the mean, (*T*-score ≤ 40). An exception of this mode of data presentation is given for the TEA, for which cut-off scores for the number of correct responses are given in the test manual, i.e., any score less than or equal to 5 indicating impaired performance. Raw scores and normative score presentation are based on individual test variables, as well as on an analysis per test, i.e., by defining impaired test performance if at least one of its variables indicates an impairment. A cumulative analysis of test performance is provided by counting the number of individuals having impairments in one or more of the tests or test variables. Finally, TOT effects are explored by calculating *T*-score differences between the first- and the second-half of each test (WAFV, TAP, TOVA), *T*-scores of changes in reaction time of correct responses over the blocks (CPT), and *T*-scores indicating the mean RT of the first two sets divided by the mean RT of the last two sets (IVA-2).

## 3. Results

**Figure 1:**
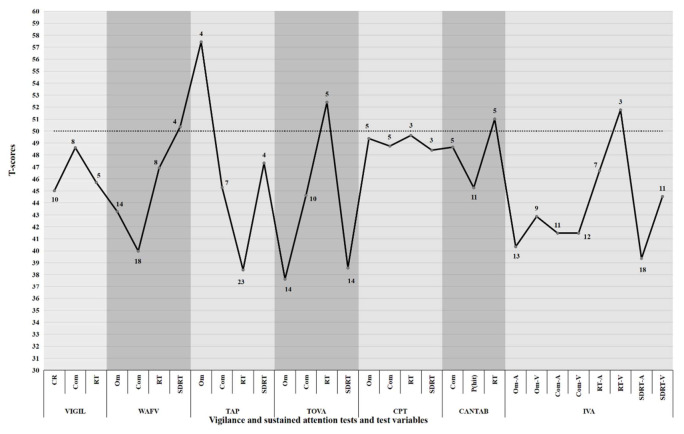
Graphical depiction of vigilance and sustained attention test performance (*T*-scores) and number of patients with ADHD showing impairment (*T* ≤ 40) in each of the test variables. Note: All raw scores were converted to *T*-scores based on test norms. Test performance scores were converted, if applicable, such that lower *T*-scores indicate worse performance for all test variables. Data labels indicate the number of patients impaired in each test variable (*T* ≤ 40). VIGIL = VIGIL of the Vienna Test System (VTS). WAFV = Perception and Attention Functions—Vigilance of the VTS. TAP = Test Battery for Attention Performance. TOVA = Test Of Variables of Attention. CPT = Conners’ Continuous Performance Test. CANTAB = Cambridge Neuropsychological Test Automated Battery—Rapid Visual Processing. IVA-2 = Integrated Visual and Auditory Continuous Performance Test. CR = Correct responses. Com = Commissions. RT = Reaction time. Om = Omissions. SDRT = Standard deviation of reaction times. P(hit) = Probability of a hit. Omi-A = Omissions, Auditory. Omi-V = Omissions, Visual. Com-A = Commissions, Auditory. Com-V = Commissions-Visual. RT-A = Reaction time, Auditory. RT-V = Reaction time, Visual. SDRT-A = Standard deviation of reaction times, Auditory. SDRT-V = Standard deviation of reaction times, Visual.

Table 2 presents a summary statistic of vigilance and sustained attention test scores for all individuals participating in this study. *T*-scores indicate whether group means are below, above, or around the performance level of typically developing individuals from the respective normative group. A *T*-score of 50 indicates the average of the normative sample, whereas performance scores are always presented in a way in which lower *T*-scores (i.e., *T*-score < 50) indicate poorer performance and higher *T*-scores (i.e., *T*-score > 50) indicate better performance. Table 2 also displays the number of individuals with ADHD showing an impairment in the respective test measure. A graphical depiction of the mean *T*-scores per test variable, in conjunction with the number of individuals scoring in the impaired range, is presented in Figure 1. An inspection of Table 2 and Figure 1 reveals considerably lower group performance of patients with ADHD when compared to test norms, as indicated by mean *T*-scores below 50 for four of the seven tests, for which *T*-scores can be calculated, including the WAFV, TAP, TOVA, and IVA-2. However, a comparison of the different tests demonstrates that not all tests applied seem to be equally sensitive in unfolding cognitive impairment in adults with ADHD, as, for example, mean *T*-scores for the VIGIL, CPT, and CANTAB tests are considerably higher than for the remaining tests applied, and lie around the mean *T*-score of 50, indicating average performance compared to normative data. This observation of differences between scores of the various vigilance and sustained attention tests is underlined by an inspection of the number of individuals showing impairment in each of the test variables, with higher numbers of impairment for the test variables of the WAFV, TAP, TOVA, and IVA-2 as compared to the other tests applied in this study.

**Figure 2:**
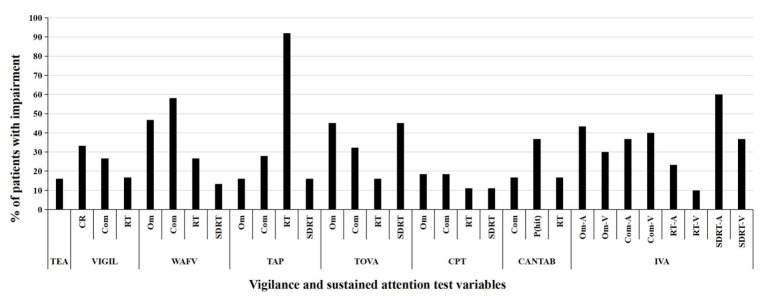
Percentage of patients showing impairment in each test variable. Note: A test variable is defined as impaired if *T*-score ≤ 40. TEA = Test of Everyday Attention. VIGIL = VIGIL of the Vienna Test System (VTS). WAFV = Perception and Attention Functions—Vigilance of the VTS. TAP = Test Battery for Attention Performance. TOVA = Test Of Variables of Attention. CPT = Conners’ Continuous Performance Test. CANTAB = Cambridge Neuropsychological Test Automated Battery—Rapid Visual Processing. IVA-2 = Integrated Visual and Auditory Continuous Performance Test. CR = Correct responses. Com = Commissions. RT = Reaction time. Om = Omissions. SDRT = Standard deviation of reaction times. P(hit) = Probability of a hit. Omi-A = Omissions, Auditory. Omi-V = Omissions, Visual. Com-A = Commissions, Auditory. Com-V = Commissions, Visual. RT-A = Reaction time, Auditory. RT-V = Reaction time, Visual. SDRT-A = Standard deviation of reaction times, Auditory. SDRT-V = Standard deviation of reaction times, Visual.

An examination of the various types of outcome variables (e.g., response time, variability of response time, or accuracy of responses) does not suggest any particular type of outcome variable to be more sensitive in revealing cognitive impairment than others (Figure 2). The outcome variable that is most sensitive in indicating cognitive impairment in the present patient sample varied per test, i.e., commission errors of the WAFV, RT of the TAP, omission errors and SDRT of the TOVA, and SDRT-A of the IVA-2. An exploration of the agreement of the various types of outcome variables is depicted in Table 3. Assuming that a specific outcome variable measures a similar construct across all tests for vigilance and sustained attention (e.g., omissions indicating mind-wandering, or commissions indicating impulsive behavior), one would expect a given examinee to either not score in the impaired range in any of the outcome variables of the same type, or to score in the impaired range in all outcome variables of the same types across the different tests. In contrast to this assumption, Table 3 shows that most individuals with ADHD score in the impaired range in some, but not all outcome variables of the same type across tests, i.e., showing impairment in a specific variable in one, two, or three tests, but not in the remaining tests. The disagreement between the various tests in the evaluation of the individual patients with ADHD is graphically depicted in Supplementary Figures S1–S4, showing marked *T*-score variations in the same test variables across tests for individual examinees.

**Figure 3:**
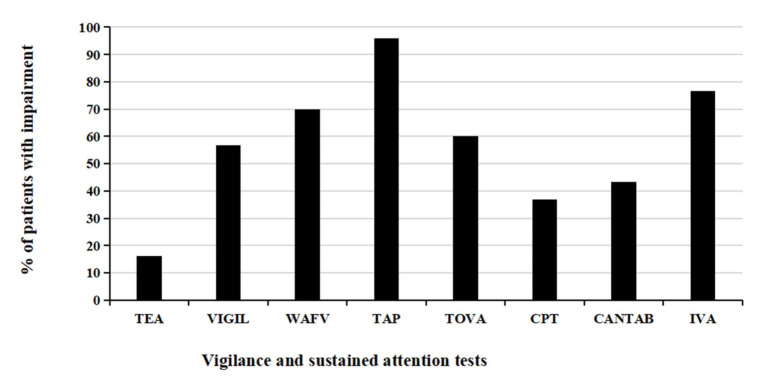
Proportion of patients with ADHD showing impairment in the various tests. Note: A test is defined as impaired if at least one test variable showing impairment. A test variable is defined as impaired when *T*-score ≤ 40. TEA = Test of Everyday Attention. VIGIL = VIGIL of the Vienna Test System (VTS). WAFV = Perception and Attention Functions—Vigilance of the VTS. TAP = Test Battery for Attention Performance. TOVA = Test Of Variables of Attention. CPT = Conners’ Continuous Performance Test. CANTAB = Cambridge Neuropsychological Test Automated Battery—Rapid Visual Processing. IVA-2 = Integrated Visual and Auditory Continuous Performance Test.

**Figure 4:**
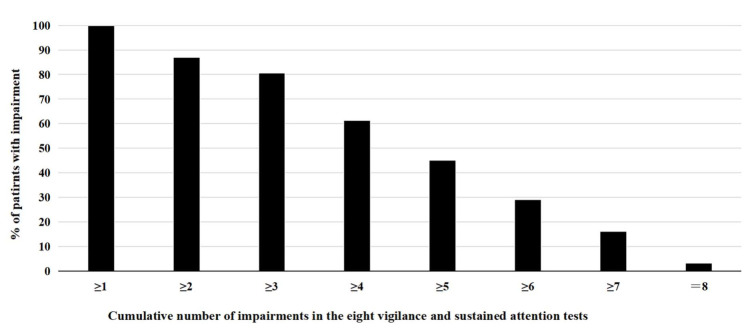
Cumulative percentage of patients with ADHD showing impairment in vigilance and sustained attention tests. Note: A test is defined as impaired if at least one test variable shows impairment. A test variable is defined as impaired when *T*-score ≤ 40.

Further, vigilance and sustained attention performance was evaluated by analyzing performance scores per test, i.e., by defining impairment if at least one test variable score of a specific test falls in the impaired range (*T* ≤ 40). Figure 3 (relative frequency of impairments per test) and Figure 4 (cumulative relative frequency of impairments per test) display the frequencies of cognitive impairment resulting from this analysis. As presented in Figure 3, more than half of the patients with ADHD exhibit impairments in the tests VIGIL, WAFV, TAP, TOVA, and IVA-2, while only a smaller proportion of patients with ADHD indicate impairments in the tests TEA, CPT, and CANTAB. Cumulative data analysis of Figure 4 shows that most patients with ADHD (>80%) have impairment in at least three of the eight tests, and still more than 60% have impairments in at least four of the eight tests.

Finally, this study addresses the issue of deterioration of performance over time and whether patients with ADHD exhibit a greater performance decrement (TOT effects) compared to typically developing individuals. Our analysis reveals no evidence for sustained attention impairments as shown by similar TOT effects in patients with ADHD compared to the respective normative groups (Table 4). *T*-score differences between the first- and the second-half of the WAFV, TAP, and TOVA reveal that the course of functioning over time (TOT effects) of patients with ADHD is not different from the course of typically developing individuals, as reflected by the norm scores. This is shown by *T*-score differences closely below or above 0, and about half of the patient group showing more, and the other half less, deterioration over time compared to the respective normative group. Similar results are derived from the CPT and IVA-2, although displayed in different metrics: mean *T*-scores indicating TOT effects are around 50, which indicates no meaningful differential change in patients with ADHD compared to the normative group of typically developing individuals. In addition, for these two tests, about half of the patients with ADHD showed more deterioration, whereas the other half showed less deterioration of performance over time.

## 4. Discussion

The present study illustratively shows broad impairments in vigilance in adults with ADHD. Impairments are evidenced on a group level by low *T*-scores (<50) in most tests and test variables, and on an individual performance level by a considerable number of patients scoring in the impaired range. *T*-scores of group performance indicated effects of one standard deviation below the mean of the respective test norms for selected variables of the WAFV, TAP, TOVA, and IVA-2 tests (see Table 2 and Figure 1 for illustration). The large effects are also reflected by individual performance scores, revealing that at least half of the patients (14–23 individuals) exhibited impairments in these aspects of functioning. These results confirm previous research, supporting the sensitivity of vigilance and sustained attention tests and advocating their use in the clinical evaluation of ADHD [6,8,11,36]. However, even though vigilance and sustained attention assessment may inform the clinician on individual cognitive strengths and weaknesses, it must be noted that its utility in the diagnostic process of ADHD has been questioned through extensive debate, as its sensitivity, specificity, and ecological validity may be insufficient to support diagnostic decision making and predict symptom severity [5,37,38,39]. Moreover, the present results demonstrate that not all tests seem to be equally sensitive, as the WAFV, TAP, TOVA, and IVA-2 tests seem to reveal more frequent and more pronounced cognitive impairment compared to the TEA, VIGIL, CPT, and CANTAB tests. The reasons for the differential sensitivity of the various tests remain unclear, as no test characteristics could be identified that are associated with conspicuous *T*-scores and more pronounced impairments. For example, an inspection of the major characteristics of the tests, such as the target rate (presentation of response demanding stimuli), test duration, or the nature of performance assessment (such as the speed or accuracy of responses), does not lead to any consistent characterization of those tests that appeared to be more sensitive in revealing cognitive impairment compared to the remaining tests applied in this study. Future research should be dedicated to disentangling test characteristics in more detail in order to identify those features that are responsible for the differential effects in revealing cognitive impairment.

Furthermore, no specific test variables stand out to be the most sensitive, as the tests varied with respect to the outcome variables indicating the largest impairment (see Figure 2), e.g., commission errors of the WAFV, RT of the TAP, omission errors and SDRT of the TOVA, and SDRT-A of the IVA-2. Results from Table 3 support this notion, as some but not all tests providing the same outcome variable in their outputs, indicate impairment. As an explanation, it can be argued that the impact of each test variable not only depends on its own nature (e.g., measurement of response times, response time variability, or number of errors), but also on the test characteristics where it is derived from (e.g., test duration, stimulus configuration, and density). As an alternative explanation, it can also be considered that the applied tests may not provide reliable indications of cognitive performance, but that intraindividual fluctuations may be responsible for performance differences on the same outcome variables [40]. This view is underlined by the marked *T*-score variations for individual examinees when inspecting the same test variable across the different tests (see Supplementary File). The disagreement in the individual evaluation across tests can be observed for the speed of responses, variability in response speed, as well as numbers of omission and commission.

The persistent and prevalent impairment in vigilance in adults with ADHD becomes particularly evident when inspecting performance scores per test and defining impairment if at least one test variable indicates impairment. With the exception of the TEA, CPT, and CANTAB, at least half, and up to almost 80% of patients with ADHD exhibited impairment when interpreting their performance per test. An analysis of the cumulative number of impairments across all eight tests demonstrates that all patients with ADHD have at least an impairment in one test, but typically show impairments in several tests, with more than 80% having at least three impairments (of a maximum of eight tests), and still more than 60% having impairments in at least four of the eight tests. The results can be seen as a confirmation of previous CPT findings in adults with ADHD, showing marked impairments in adults with ADHD on a group level, but insufficient sensitivity and specificity for diagnostic decision-making and for the prediction of self-reported symptom severity [38]. The large proportions of patients with ADHD having multiple impairments supports the convergent validity of the tests and the related underlying construct. However, assuming all tests applied in this study measure the same cognitive construct, one would expect that patients with ADHD would either have no impairment in any of the tests or show impairments in all tests applied. Intraindividual day-to-day fluctuations in symptoms and performance could account for this effect, which may seriously question the validity of a one-time neuropsychological assessment of patients with ADHD and emphasizes the need for a more extensive diagnostic process with repeated symptom assessments [40].

Further, our analysis of TOT effects and the comparison to test norms arrived at no evidence for the existence of sustained attention deficits in adults with ADHD. The lack of sustained attention impairments, as defined by a greater decrement in task performance over time compared to typically developing individuals, underlines the findings of Tucha and colleagues (2009) who reported no sustained attention deficits in children and adults with ADHD in the vigilance test of the TAP. The present study extends previous research in this field by demonstrating a lack of sustained attention deficits in a broad range of measures. This study gives more clarity to a, so far, unresolved question as to whether attention deficits are present and remain stable from the beginning of an attention assessment [41,42], or unfold particularly over test progression in tests with a longer administration time [7,8,43]. Given the broad battery of attention tests in this study, which uniformly speaks against the existence of ADHD characteristic decrements in task performance, we conclude that attention deficits in adults with ADHD may not depend on task duration but unfold also in short-term tasks requiring attentional control. As a consequence, one may infer that widely recommended behavioural cognitive strategies for the management of ADHD, such as scheduling cognitive tasks, breaking down longer tasks in to short time units, and introducing structured breaks in long meetings, may support motivational issues in individuals with ADHD [44], but may equally benefit individuals with and without ADHD, in terms of protecting against cognitive performance decrements.

Finally, next to the adult population, vigilance and sustained attention tests are popular instruments in the diagnostic evaluation of children with ADHD. CPTs in children with ADHD received broad attention from research and are controversially discussed regarding their sensitivity, specificity, ecological validity, and usefulness in treatment planning and evaluation [41,45,46]. As the present study was designed for adults, and no conclusions can be drawn for children and adolescents, future research using this or comparable design is needed in order to add evidence on the use of vigilance and sustained attention tests for the assessment of ADHD in childhood. A comparison between tests and test variables, and possibly a repeated assessment on several occasions, may better reflect the complexity and heterogeneity of ADHD from childhood to adulthood.

## 5. Limitation and Future Directions

The present study must be seen in light of several limitations. Because a repeated assessment of patients with ADHD with a battery of long-lasting and exhausting neuropsychological tests is difficult to achieve, this study made use of a relatively small and selective sample. The present results should, therefore, be regarded as preliminary, pending replication in larger samples. Larger samples allow for the consideration of disease characteristics, including symptom presentation, symptom severity, comorbidity, and medication status, and give more reliable estimates about the proportion of people with impairments in the various aspects of functioning. Larger samples would be particularly suited to examining the contribution of comorbid psychopathologies, such as depression, anxiety, or substance abuse, on vigilance and sustained attention deficits in adults with ADHD.

Second, this study lacked a comparison group of typically developing individuals who performed the battery of tests in the same design. Such a comparison group would serve as a common standard to which all tests can be compared, and would facilitate a cross-comparison of the sensitivity of all tests and test variables. Such a control group would be particularly beneficial because all test performances of patients with ADHD would be compared to the same group of typically developing individuals who serve as a common metric in test data interpretation. For clinical application and differential diagnostic purposes, this study would further benefit from performance data of clinical comparison groups who are commonly seen in the same referral context, such as patients with substance abuse, anxiety, mood disorder, or personality disorder. Clinical comparison groups who performed the same test battery would provide crucial information regarding the utility of the applied tests for differential diagnostics. Even though a carefully matched comparison group would be preferred, the validity of the present findings may be safeguarded by the quality and representativeness of test norms as provided by the test publishers. The advantage of considering test norms in this study is that patients with ADHD are compared to a large representative group of typically developing individuals, and the performance of each individual patient with ADHD can be compared to a cohort of typically developing individuals of the same age and/or similar other characteristics. A convenience sample of typically developing individuals specifically recruited and assessed for the purpose of this study would presumably be smaller in size, and may therefore represent the population of typically developing individuals less well.

Third, performance differences between tests in the present study could be attributed either to differences in test characteristics or intraindividual fluctuations of performance over time. This issue cannot be fully answered due to the study design of performing different tests on different assessment days. A reassessment of the same tests on different assessment days would give further information on the reliability of the various vigilance and sustained attention tests, and possible intraindividual fluctuations of performance.

Fourth, this study used different metrics for the calculation of TOT effects for the various tests, which may distort the determination of sustained attention deficits for some of the tests, and may also confound a valid comparison of TOT effects between tests and test variables. A thorough analysis of TOT effects across all tests for vigilance and sustained attention may require the definition of equally long time blocks within each test and the determination of the test output per time block. Such an analysis may be difficult to achieve, not only because of differences in test duration, but also because of differences in stimulus frequency and density.

## 6. Conclusions

Although the results require independent replication in larger samples, preliminary conclusions can be drawn that may be particularly relevant for clinical practice and the neuropsychological evaluation of adult ADHD. First, this study suggests that vigilance and sustained attention tests may be useful tools to reveal cognitive impairments in adults with ADHD, particularly the WAFV, TAP, TOVA, and IVA-2 tests. Second, clinicians may be advised to consider all variables of a test as cognitive impairments in adult ADHD do not seem to be bound to specific variable types. Third, this study adds evidence to the notion that cognitive impairment in adults with ADHD unfolds best in summary scores over the entire test duration instead of in a differential investigation of TOT effects. Finally, we suggest and hypothesize, pending replication in larger samples, that the neuropsychological assessment of adults with ADHD using cognitive performance tests may benefit from a reassessment on a different day, in order to adequately take intraindividual fluctuations and limitations regarding test reliability into account.

## Figures and Tables

**Table 1:** Characteristics and symptom scores of patients with ADHD.

	Patients with ADHD (*n* = 31)					
Gender (Male/Female)	17/14					
Education (1/2/3/4/5/6/7) ^a^	0/2/3/5/2/5/14					
	Min	Max	Median	Mean	SD	% with Impairment ^f^
Age (in years)	21	64	34	37.29	12.28	-
Childhood ADHD symptoms ^b^	21	67	41	42.33	11.78	-
Current ADHD symptoms ^c^						
-Inattention	2	26	17	16.00	5.92	-
-Hyperactivity/Impulsivity	3	23	12	12.78	5.65	-
-Symptoms total	7	47	31	28.78	10.18	-
Depressive symptoms ^d^	0	53	10	12.87	12.50	-
Functional impairments ^e^						
-Family	0	2.50	1.06	1.07	0.74	64.29
-Work	0	2.50	1.40	1.25	0.82	69.23
-School	0.18	2.78	1.45	1.42	0.64	84.00
-Life skills	0.17	2.55	1.38	1.27	0.58	92.31
-Self-concept	0	2.80	1.50	1.36	0.97	64.29
-Social	0	2.78	0.89	0.97	0.76	57.69
-Risk	0	2.57	0.51	0.68	0.57	51.85

Note: ^a^ Education (1/2/3/4/5/6/7) = No formal qualification/basic school education/basic school education and training/secondary school leaving qualification/higher secondary school education/university entrance qualification/university entrance qualification/completed training or study program. ^b^ Measured with the Wender-Utah Rating scale, short version (WURS-K); scores were missing in four cases. ^c^ Measured with the Conners’ Adult ADHD Rating Scale (CAARS). ^d^ Measured with the Beck Depression Inventory (BDI–2). ^e^ Measured with the Weiss Functional Impairment Rating Scale (WFIRS). ^f^ Percentage of patients with ADHD who indicated impairment in each of the functional domains. For each patient, any domain that with at least two items scored as 2, or one item scored 3, or a mean score > 1.5, is defined as impaired.

**Table 2:** Vigilance and sustained attention test performances of patients with ADHD.

	Patients with ADHD (*n* = 31)						
	Min	Max	Median	Mean	SD	*T*-Score ^a^	N with Impairment ^b^
TEA ^c^							
Number correct	4	7	7	6.63	0.85	-	5
VIGIL ^d^							
Correct responses	10	32	25.50	25.10	5.21	45.01	10
Commissions	0	194	4.50	17.87	37.94	48.61	8
RT ^e^	525	1174	683	689	141	45.70	5
WAFV ^f^							
Omissions	0	36	4	6.27	8.13	43.27	14
Commissions	0	57	5.50	9.87	12.42	39.97	18
RT ^e^	382	783	465	491	88.79	46.92	8
SDRT ^g^	1.1	2.82	1.24	1.29	0.31	50.35	4
TAP ^h^							
Omissions	0	21	2.50	4.77	6.11	57.44	4
Commissions	0	222	5	28.10	58.28	45.28	7
RT ^e^	636	1867	865	905	210	38.40	23
SDRT ^g^	72	568	195	225	125	47.32	4
T.O.V.A. ^i^							
Omissions	0	67	2	7.42	13.94	37.63	14
Commissions	0	71	10	13.81	14.33	44.62	10
RT ^e^	279	764	355	374	90.45	52.40	5
SDRT ^g^	60	332	94	113	61.96	38.56	14
CPT ^j^							
Omissions	0	26.4	0.69	2.28	5.14	49.37	5
Commissions	5.56	70.83	31.94	29.78	18.06	48.74	5
RT ^e^	340	556	415	419	58.62	49.63	3
SDRT ^g^	59	312	103	111	50.30	48.40	3
CANTAB ^k^							
Commissions	0	7	1	1.6	1.77	48.65	5
P(hit) ^l^	0.26	0.96	0.56	0.58	0.18	45.26	11
RT ^e^	337	964	465	481	127	51.00	5
IVA-2 ^m^							
Auditory omissions	91.10	100	100	97.26	3.49	40.34	13
Visual omissions	71.10	100	100	96.22	7.06	42.86	9
Auditory commissions	78.70	100	96	95.07	4.29	41.47	11
Visual commissions	86.20	100	95.40	94.31	4.19	41.47	12
Auditory RT ^e^	57	810	602	585	159	46.69	7
Visual RT ^e^	64	976	414	433	152	51.76	3
Auditory SDRT ^g^	59.40	86	74.55	74.93	6.36	39.35	18
Visual SDRT ^g^	63.20	85	75.70	75.69	5.49	44.53	11

Note: ^a^ All raw scores were converted to *T*-scores based on test norms. Test performance scores were converted, if applicable, such that lower *T*-scores indicate worse performance for all test variables. ^b^ Number of patients with ADHD with a *T*-score ≤ 40. ^c^ Test of Everyday Attention. No *T*-Scores can be derived from test scores. Cut-off scores (≤5) indicating impairment were derived from the test manual. ^d^ VIGIL (VTS). ^e^ Reaction time of correct responses in milliseconds. ^f^ Perception and Attention Functions—Vigilance (VTS). ^g^ Standard deviation of reaction times of correct responses. ^h^ Test Battery for Attention Performance. ^i^ Test Of Variables of Attention. ^j^ Conners’ Continuous Performance Test. ^k^ Cambridge Neuropsychological Test Automated Battery—Rapid Visual Processing. ^l^ Probability of a hit. ^m^ Integrated Visual and Auditory Continuous Performance Test.

**Table 3:** Proportion of patients with ADHD showing impairments in no, one, or more than one test regarding the corresponding test variable.

	Number of Tests Providing the Respective Test Variable							
	0	1	2	3	4	5	6	7
Omissions ^a^	29.0%	19.35%	19.35%	25.8%	0	6.5%	-	-
Commissions ^b^	22.5%	16.1%	25.8%	9.7%	12.9%	6.5%	6.5%	0
RT ^c^	12.9%	32.3%	29.0%	16.1%	6.5%	3.2%	0	0
SDRT ^d^	25.8%	25.8%	35.4%	6.5%	6.5%	0	-	-

Note: The percentage rates indicate the proportion of patients with ADHD who show no, one, or more than one impairment in the corresponding test variable when inspecting all tests for vigilance and sustained attention. ^a^ Five tests provide omission errors in their test output, including WAFV, TAP, TOVA, CPT, and IVA-2. ^b^ Seven tests provide commission errors in their test output, including VIGIL, WAFV, TAP, TOVA, CPT, CANTAB, and IVA-2. ^c^ Seven tests provide RT in their test output, including VIGIL, WAFV, TAP, TOVA, CPT, CANTAB, and IVA-2. ^d^ Five tests provide SDRT in their test output, including WAFV, TAP, TOVA, CPT, and IVA-2.

**Table 4:** *T*-scores and *T*-scores differences indicating time-on-task effects.

Patients with ADHD (*n* = 31)					
	Min	Max	Mean	SD	N Showing More Deterioration than the Normative Group
WAFV ^a^					
Difference in omissions	−12.10	21.90	2.87	8.41	12
Difference in commissions	−14.00	27.90	3.81	11.01	17
Difference in RT	−13.90	14.10	1.60	7.13	17
Difference in SDRT	−35.20	23.60	0.72	11.41	17
TAP ^a^					
Difference in omissions	−17.00	20.00	−1.84	8.50	4
Difference in commissions	−20.00	20.00	0.72	8.81	17
Difference in RT	−12.00	3.00	−2.84	2.68	2
Difference in SDRT	−13.00	20.00	0.40	8.01	11
T.O.V.A. ^a^					
Difference in omissions	−33.30	30.70	3.11	13.04	11
Difference in commissions	−26.70	28.70	5.80	11.59	19
Difference in RT	−15.30	6.00	−4.73	5.63	5
Difference in SDRT	−34.70	19.30	−2.66	11.45	7
CPT ^b^					
Block change in RT	17.00	68.00	48.40	11.76	13
IVA-2 ^c^					
Auditory stamina	26.70	80.00	49.28	11.85	15
Visual stamina	19.30	73.30	47.71	11.77	17

Note: ^a^ For WAFV, TAP, and T.O.V.A.: *T*-score differences are calculated by the *T*-score of the first-half minus the *T*-score of the second-half per test variable. Mean difference *T*-scores > 0 indicate better performance in the first-half of the test than the second-half. ^b^ *T*-score of the change in reaction time of correct responses over the blocks (test progression). *T*-scores < 50 indicate better performance in earlier blocks than later blocks. ^c^ Stamina is calculated by the mean RT of the first two sets divided by the mean RT of the last two sets. *T*-scores < 50 indicate better performance in the first-half of the test than the second-half.

## Data Availability

The data that support the findings of this study are available from the corresponding author upon reasonable request.

## Supplementary Materials

The following supporting information can be downloaded at: https://www.mdpi.com/article/10.3390/ijerph19095216/s1, Figure S1: T-scores of reaction time variables for each individual patient with ADHD; Figure S2: T-scores of the standard deviation of reaction time variables for each individual patient with ADHD; Figure S3: T-scores of omission error variables for each individual patient with ADHD; Figure S4: T-scores of commission error variables for each individual patient with ADHD.
