# Supplementary material for: It Takes Time: Vigilance and Sustained Attention Assessment in Adults with ADHD
Source: Int J Environ Res Public Health. 2022 Apr 25;19(9):5216. doi: 10.3390/ijerph19095216 (PMC9102294; doi:10.3390/ijerph19095216)
Supplement: Supplementary file 1 [file ijerph-19-05216-s001.zip › ijerph-1683015-supplementary.pdf]

Supplementary file

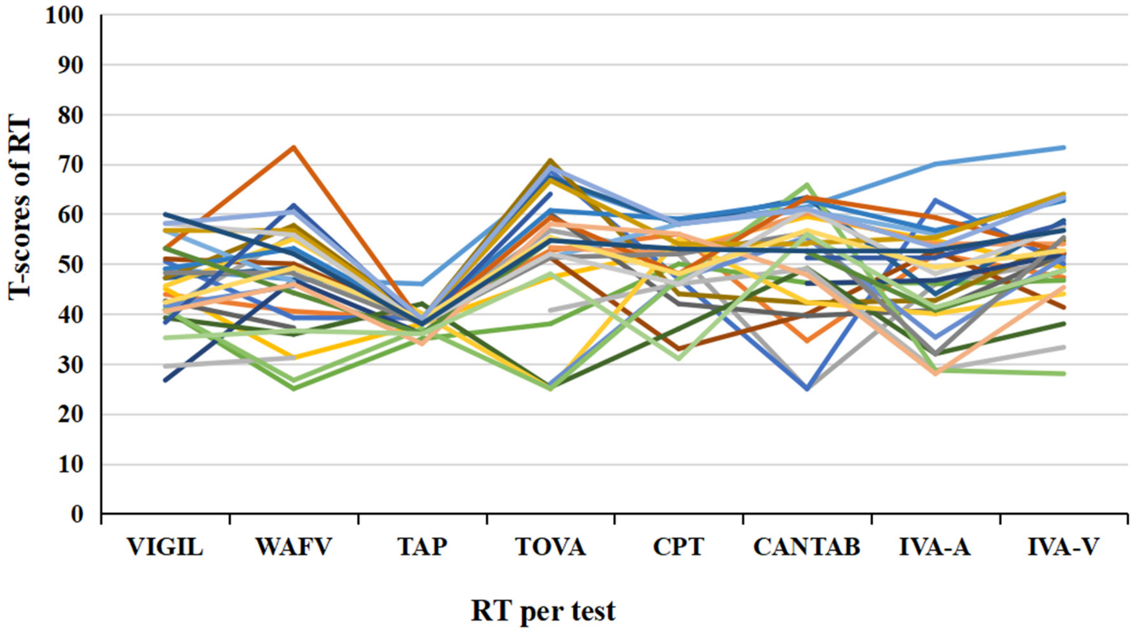

Supplementary Figure S1: *T*-scores of reaction time variables for each individual patient with ADHD. *Notes:* Each line represents one participant. RT = Reaction time, VIGIL = VIGIL of the Vienna Test System (VTS), WAFV = Perception and Attention Functions—Vigilance of the VTS, TAP = Testbattery for Attention Performance, TOVA = Test Of Variables of Attention, CPT = Conners' Continuous Performance Test, CANTAB = Cambridge Neuropsychological Test Automated Battery—Rapid Visual Processing, IVA-A = Integrated Visual and Auditory Continuous Performance Test (IVA-2)—Auditory, IVA-V = Integrated Visual and Auditory Continuous Performance Test (IVA-2)—Visual.

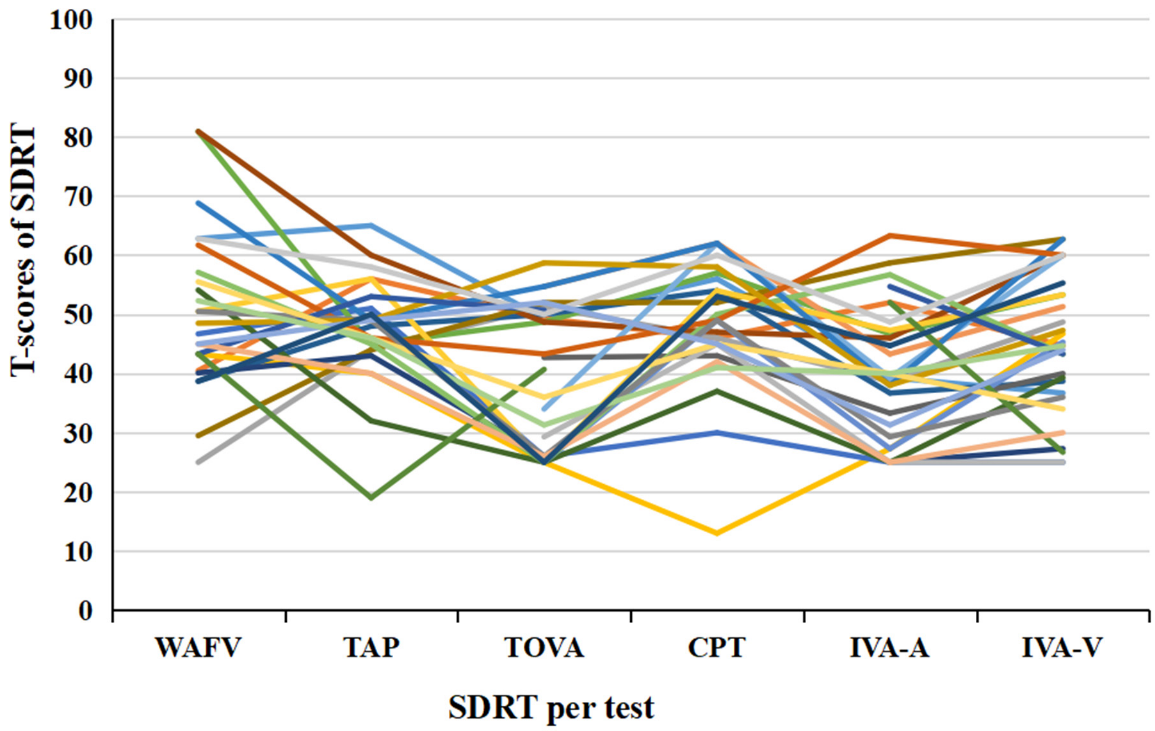

Supplementary Figure S2: *T*-scores of the standard deviation of reaction time variables for each individual patient with ADHD. *Notes:* Each line represents one participant. SDRT = standard deviation of reaction time, VIGIL = VIGIL of the Vienna Test System (VTS), WAFV = Perception and Attention Functions—Vigilance of the VTS, TAP = Testbattery for Attention Performance, TOVA = Test Of Variables of Attention, CPT = Conners' Continuous Performance Test, CANTAB = Cambridge Neuropsychological Test Automated Battery—Rapid Visual Processing, IVA-A = Integrated Visual and Auditory Continuous Performance Test (IVA-2)—Auditory, IVA-V = Integrated Visual and Auditory Continuous Performance Test (IVA-2)—Visual.

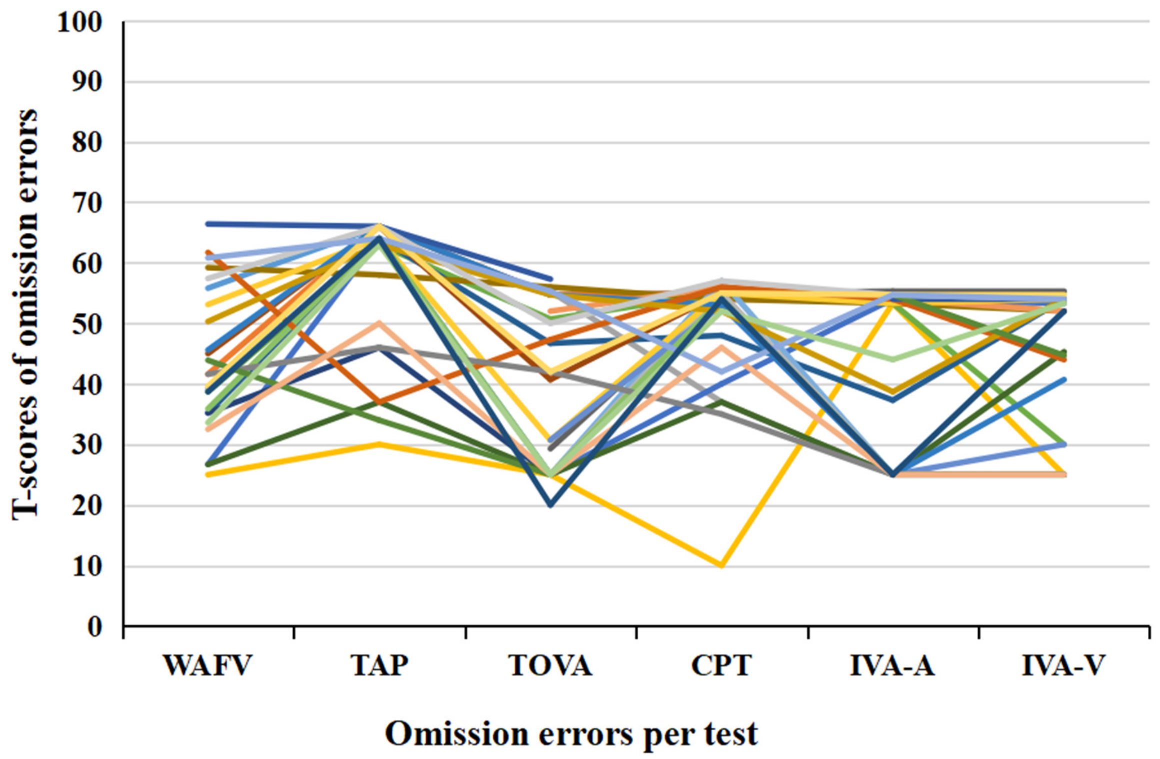

Supplementary Figure S3: *T*-scores of omission error variables for each individual patient with ADHD. *Notes:* Each line represents one participant. VIGIL = VIGIL of the Vienna Test System (VTS), WAFV = Perception and Attention Functions—Vigilance of the VTS, TAP = Testbattery for Attention Performance, TOVA = Test Of Variables of Attention, CPT = Conners' Continuous Performance Test, CANTAB = Cambridge Neuropsychological Test Automated Battery—Rapid Visual Processing, IVA-A = Integrated Visual and Auditory Continuous Performance Test (IVA-2)—Auditory, IVA-V = Integrated Visual and Auditory Continuous Performance Test (IVA-2)—Visual.

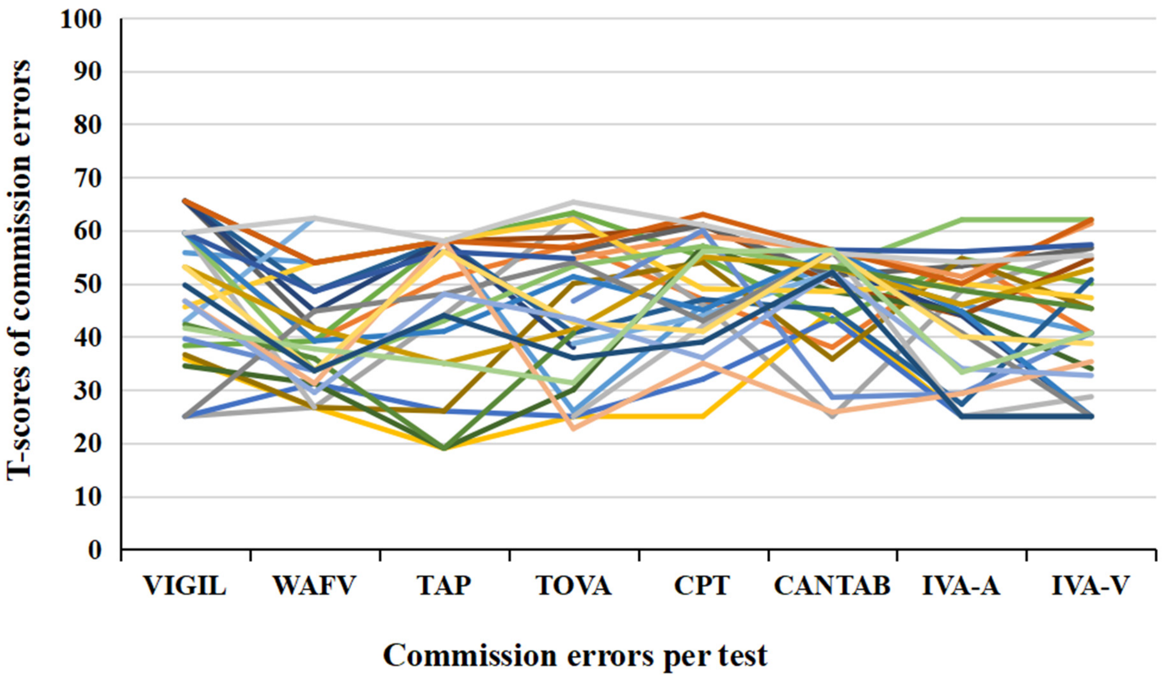

Supplementary Figure S4: T-scores of commission error variables for each individual patient with ADHD. *Notes:* Each line represents one participant. VIGIL = VIGIL of the Vienna Test System (VTS), WAFV = Perception and Attention Functions—Vigilance of the VTS, TAP = Testbattery for Attention Performance, TOVA = Test Of Variables of Attention, CPT = Conners' Continuous Performance Test, CANTAB = Cambridge Neuropsychological Test Automated Battery—Rapid Visual Processing, IVA-A = Integrated Visual and Auditory Continuous Performance Test (IVA-2)—Auditory, IVA-V = Integrated Visual and Auditory Continuous Performance Test (IVA-2)—Visual.
